# Selenomethionine as a dual-mechanism ferroptosis inhibitor: selenium-supply-driven GPX4 biosynthesis beyond transsulfuration and reductive-capacity-mediated ROS scavenging independent of GPX4 activity

**DOI:** 10.1038/s41419-026-08466-x

**Published:** 2026-02-14

**Authors:** Chaoyi Xia, Xue Sun, Junyi Shao, Jingshu Min, Chong Wei, Feiyang Zhao, Caiyun Fu, Qiang Zhang

**Affiliations:** 1 Zhejiang Provincial Engineering Research Center of New Technologies and Applications for Targeted Therapy of Major Diseases, Zhejiang Provincial Key Laboratory of Drug Discovery and Development for Metabolic Diseases, College of Life Sciences and Medicine, Zhejiang Sci-Tech University, Hangzhou, Zhejiang Province China; 2 Postgraduate Training Base Alliance of Wenzhou Medical University (Wenzhou People’s Hospital), Wenzhou, China; 3 Department of Biophysics, and Second Affiliated Hospital, Zhejiang University School of Medicine, Hangzhou, Zhejiang Province China

**Keywords:** Cell death, Drug screening

## Abstract

Ferroptosis is an iron-dependent form of nonapoptotic cell death driven by lipid peroxidation. The selenium-dependent glutathione peroxidase 4 (GPX4) serves as the central regulator of ferroptosis through enzymatic reduction of phospholipid hydroperoxides (PLOOH). While GPX4 remains the canonical ferroptosis suppressor, whether alternative regulatory axes exist beyond this selenoprotein-mediated pathway remains unclear. In the present study, we identified selenomethionine as a novel resister of ferroptosis induced by RSL3 through screening FDA drug library and natural product library. Mechanistically, selenomethionine serves as a selenium donor for GPX4 biosynthesis beyond the transsulfuration pathway. The anti-ferroptosis activity of selenomethionine persists even after CRISPR-mediated GPX4 knockout, revealing a GPX4-independent mechanism that relies on direct redox modulation via selenium-mediated reactive oxygen species (ROS) scavenging. Significantly, selenomethionine administration effectively mitigated cisplatin-induced acute kidney injury in vivo by suppressing ferroptosis. This work establishes selenomethionine as a unique dual-mechanism ferroptosis suppressor that simultaneously modulates enzymatic antioxidant defense through GPX4 biosynthesis and non-enzymatic radical trapping via selenium-mediated redox cycling, providing new insights into therapeutic strategies for ferroptosis-related pathologies.

## Introduction

Ferroptosis, a type of regulated cell death triggered by iron-dependent lipid peroxidation in the plasma membrane or membrane organelles [1–4]. It is morphologically, biochemically, and genetically distinct from other forms of cell death, such as apoptosis, necroptosis, pyroptosis, and cuproptosis [5–7]. Although ferroptosis has been reported to be involved in shaping embryonic tissue [8]. its physiological function remains unclear. Excessive ferroptosis is involved in the pathogenesis of multiple diseases that have been studied extensively, including neurodegenerative diseases and ischemia-reperfusion injury [9, 10]. Consequently, the urgent need to identify druggable targets for ferroptosis inhibition has emerged as a central focus in developing therapeutic strategies against ferroptosis-associated pathologies.

Unrestricted lipid peroxidation has been identified as a crucial hallmark of ferroptosis, cells have evolved several highly efficient defense mechanisms against uncontrolled lipid peroxidation [11]. Among these, selenium-dependent glutathione peroxidase 4 (GPX4) is crucial for preventing ferroptosis by detoxifying phospholipid hydroperoxide into the corresponding phospholipid alcohol at the expense of glutathione (GSH) [12–15]; Ferroptosis suppressor protein-1 (FSP1) is an oxidoreductase that reduces ubiquinone (CoQ) to ubiquinol (CoQH_2_) [16, 17] or reduces oxidized hydroxyoestradiols [18]; There are also guanosine triphosphate cyclohydrolase-1 (GCH1) /tetrahydrobiopterin (BH4) systems [19], dihydroorotate dehydrogenase (DHODH)/CoQ_10_ system [20], and 7-Dehydrocholesterol (7-DHC) [21, 22], which effectively shields phospholipids from autoxidation and subsequent fragmentation. In addition, cells harness exogenous vitamin K by a non-canonical vitamin K cycle to inhibit overwhelming lipid peroxidation [23]. Fundamentally, at the core of cellular antioxidant defense lies an evolutionarily conserved selenocysteine-cysteine redox axis, which serves as the primary defense axis against iron-catalyzed lipid peroxidation cascades and ferroptosis execution [24]. The trace element selenium (Se) exerts its essential role as the 21st amino acid, selenocysteine (Sec) [25]. Sec is incorporated into the UGA codon to synthesize selenoproteins, which involves a highly complex and energetically costly process. During this process, selenide is phosphorylated into H_2_SePO_3_^-^ by selenophosphate synthetase 2 (SEPHS2) and subsequently selenocysteyl-transfer RNA for Sec (Sec-tRNA^[Ser]Sec^) is biosynthesized from reactive H_2_SePO_3_^-^ on its cognate tRNA^[Ser]Sec^ through a series of enzymatic reactions [25]. Finally, Sec-tRNA^[Ser]Sec^ is inserted into the corresponding selenoprotein, which requires the sec-insertion sequence (SECIS) element of the 3′-untranslated region of mRNAs [25]. Selenomethionine, as a naturally occurring amino acid containing selenium, it is an important source of selenium for mammalian life. However, it is still unclear how selenomethionine provides selenium for GPX4 synthesis and whether it can participate in fighting against ferroptosis.

In this study, through comprehensive screening of FDA drug library and natural product library, we identified selenomethionine as a novel ferroptosis inhibitor. Our mechanistic investigations revealed that selenomethionine serves as a selenium donor for GPX4 biosynthesis beyond the transsulfuration pathway. Notably, CRISPR/Cas9-mediated GPX4 knockout experiments demonstrated that selenomethionine retains its anti-ferroptosis activity in GPX4-deficient cells, suggesting the existence of GPX4-independent mechanisms. Further studies established that selenomethionine exerts its protective effects through direct scavenging of intracellular ROS. The therapeutic potential of this dual mechanism was validated in a cisplatin-induced acute kidney injury model, where selenomethionine administration significantly attenuated renal damage through ferroptosis inhibition. These findings not only elucidate the molecular basis of selenomethionine-mediated selenium donation for GPX4 synthesis but also reveal its GPX4-independent antioxidant properties, thereby closing a gap in our understanding of how selenomethionine provides selenium for GPX4 synthesis and inhibits ferroptosis.

## Results

### Identification of selenomethionine as a suppressor of ferroptosis

**Fig. 1:**
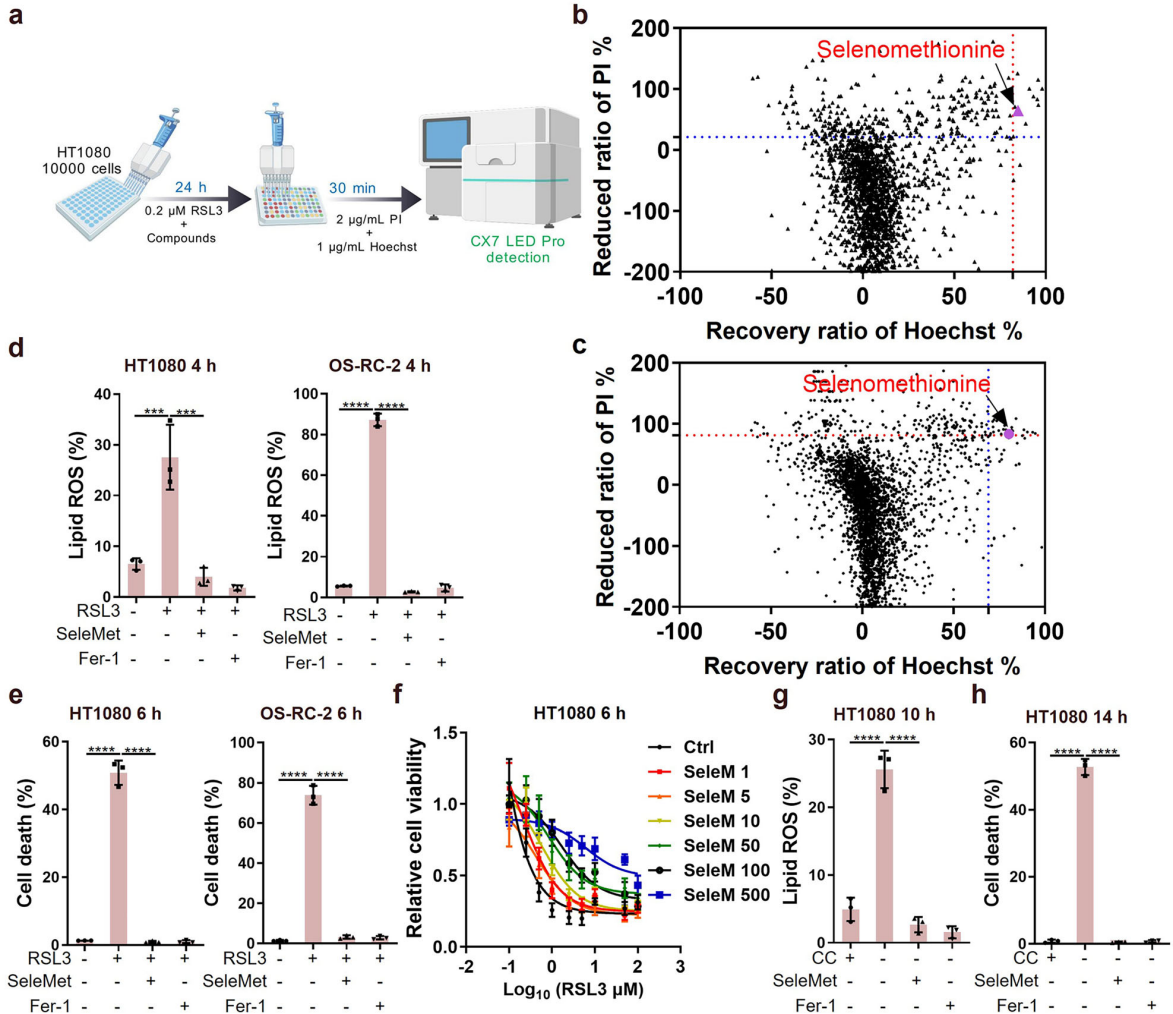
Identification of selenomethionine as a suppressor of ferroptosis. **a** Schematic of the small molecule screening strategy. **b** The small molecule screening results were exhibited as the reduced ratio of PI and the recovery ratio of Hoechst in small molecule-treated cells versus vehicle-treated cells. After cell seeding, the HT1080 cells were incubated with the FDA drug (2625) or DMSO and 0.2 μM RSL3 for 24 h. Then, the cell was measured with PI and Hoechst using CX7 LED Pro. **c** The small molecule screening results were exhibited as the reduced ratio of PI and the recovery ratio of Hoechst in small molecule-treated cells versus vehicle-treated cells. After cell seeding, the HT1080 cells were incubated with the natural product (3651) or DMSO and 0.2 μM RSL3 for 24 h. Then, the cell was measured with PI and Hoechst using CX7 LED Pro. **d**, **e** HT1080 and OS-RC-2 cells were treated with RSL3 (2 μM) or selenomethionine (SeleMet) (100 μM) as indicated for 4–6 h (*n* = 3). **d** The lipid ROS levels were determined using flow cytometry (HT1080 and OS-RC-2 for 4 h) (*n* = 3). **e** The cell death levels were quantified using flow cytometry (HT1080 and OS-RC-2 for 6 h) (*n* = 3). **f** The cell viability of HT1080 cells that were treated with RSL3 and SeleMet (100 μM) for 6 h (*n* = 3). **g**, **h** HT1080 cells are cultured in ± CC (cystine) medium treated with SeleMet (100 μM) or Ferrostatin-1 (Fer-1, 10 μM) as indicated for 10-14 h (*n* = 3). **g** The lipid ROS levels were determined using flow cytometry (HT1080 for 10 h) (*n* = 3). **h** The cell death levels were quantified using flow cytometry (HT1080 for 14 h) (*n* = 3). CC, cystine, 0.4 mM; Fer-1, Ferrostatin-1, 10 μM. Data were represented as mean ± SD with *P* values determined by one-way ANOVA (d, e, g, and h). ***P < 0.001; ****P < 0.0001; ns, nonsignificant.

The ferroptosis defense system mediated by exogenous vitamin K through FSP1 represents a unique mechanism. To explore whether there are other endogenous-exogenous synergistic systems capable of efficiently suppressing ferroptosis, we screened the FDA drug library and natural product library in ferroptosis induced by the GPX4 inhibitor RSL3, to identify new suppressors through utilizing high-throughput screening based on high-content screening and image analysis (Fig. 1a). Using ferrostatin-1 (fer-1) as the analytical benchmark, screening identified selenomethionine as a ferroptosis inhibitor in both the FDA drug library and natural product library (Fig. 1b, c). To validate these findings, we employed multiple ferroptosis-specific assays. Using BODIPY-C11 581/591 for lipid ROS detection and propidium iodide (PI) for cell death assessment, we demonstrated that selenomethionine significantly attenuated RSL3-induced lipid ROS accumulation and cell death in both HT1080 and OS-RC-2 cell lines (Fig. 1d, e). Complementary CCK-8 assays confirmed the protective effects of selenomethionine on cell viability in HT1080 cells (Fig. 1f). Furthermore, selenomethionine effectively suppressed ferroptosis induced by cystine deprivation in HT1080 cells, as evidenced by reduced lipid ROS and decreased cell death (Fig. 1g, h). Given that ferroptosis induction through either GPX4 inhibition (RSL3) or cystine depletion represents the two principal experimental paradigms in ferroptosis research, our comprehensive data establish selenomethionine as a universal ferroptosis suppressor with consistent efficacy across different induction mechanisms.

### Involvement of selenomethionine in GPX4 synthesis and ferroptosis inhibition beyond transsulfuration

**Fig. 2:**
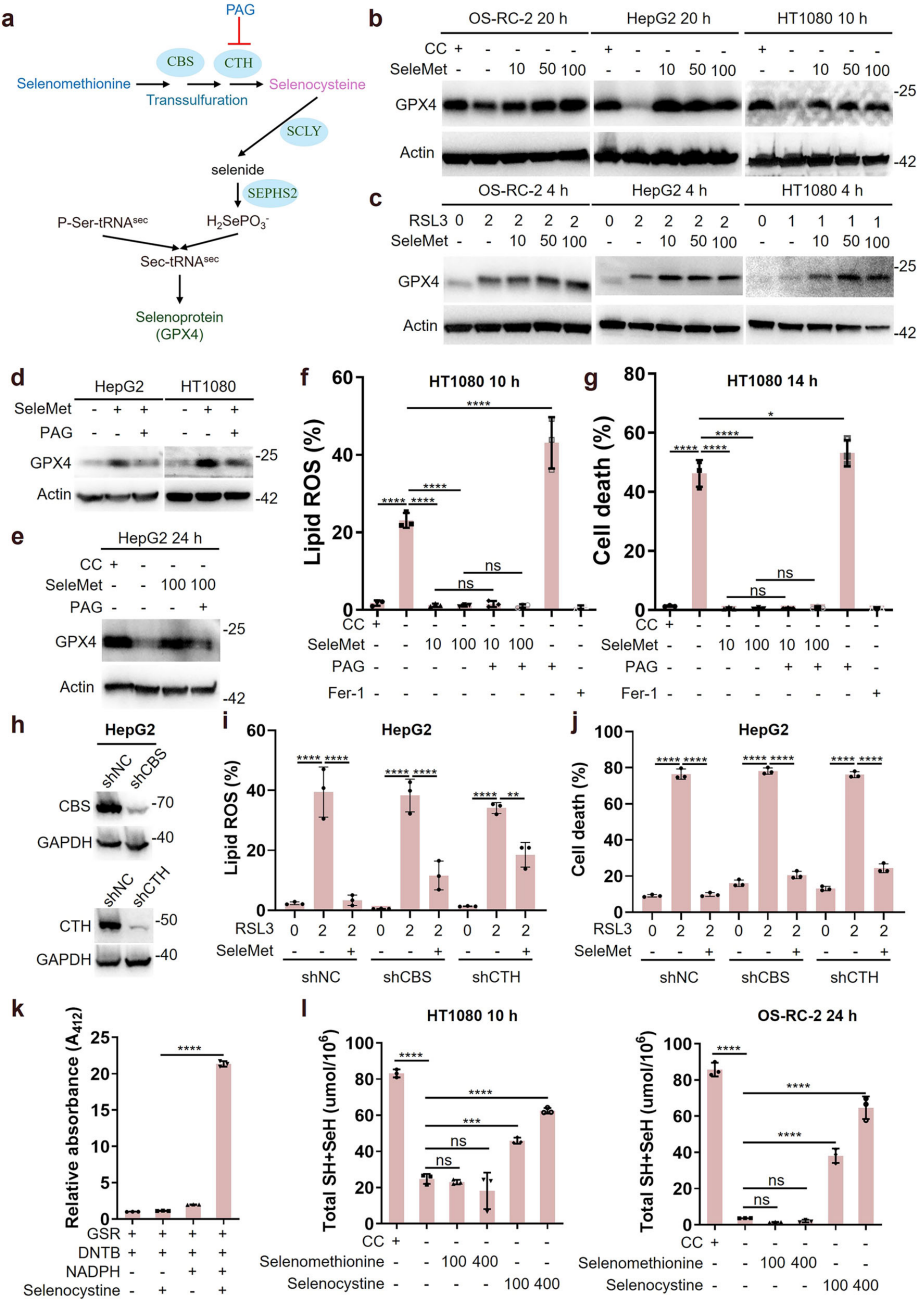
Involvement of selenomethionine in GPX4 synthesis and ferroptosis inhibition beyond transsulfuration. **a** Schematic of metabolic processes of selenium containing amino acids. **b** The protein levels of the GPX4 in OS-RC-2, HepG2, and HT1080 cells are cultured in ± CC (cystine) medium treated with SeleMet were measured by western blot (WB). **c** The protein levels of the GPX4 in OS-RC-2, HepG2, and HT1080 cells are cultured in medium treated with RSL3 or SeleMet were measured by WB. **d** The protein levels of the GPX4 in HepG2 and HT1080 cells are cultured in medium treated with SeleMet or DL-Propargylglycine (PAG, 100 μM) were measured by WB. **e** The protein levels of the GPX4 in HepG2 cells are cultured in ± CC (cystine) medium treated with SeleMet or PAG were measured by WB. **f**, **g** HT1080 cells are cultured in ± CC (cystine) medium treated with SeleMet, PAG, or fer-1 as indicated for 10-14 h (*n* = 3). **f** The lipid ROS levels were determined using flow cytometry (HT1080 for 10 h) (*n* = 3). **g** The cell death levels were quantified using flow cytometry (HT1080 for 14 h) (*n* = 3). **h** The protein levels of the CBS and CTH in HepG2 cells treated with shCBS and shCTH were measured by WB. **i**, **j** HepG2 (shNC, shCBS, and shCTH) cells are treated with RSL3 or SeleMet as indicated for 4-6 h (*n* = 3). **i** The lipid ROS levels were determined using flow cytometry (for 4 h) (*n* = 3). **j** The cell death levels were quantified using flow cytometry (for 6 h) (*n* = 3). **k** Analysis of the transformation from selenocystine to selenocysteine using DNTB. The transformation reaction is catalyzed by GSR and utilizes NADPH as the reducing force. **l** HT1080 and OS-RC-2 cells are cultured in ± CC (cystine) medium treated with selenomethionine or selenocystine as indicated for 10–24 h, then the total SH+SeH is measured by DNTB (*n* = 3). CC, cystine, 0.4 mM; Fer-1, Ferrostatin-1, 10 μM; PAG, DL-Propargylglycine. Data were represented as mean ± SD with *P* values determined by one-way ANOVA (f, g, i, j, k, and l). *P < 0.05; **P < 0.01; ***P < 0.001; ****P < 0.0001; ns, nonsignificant.

GPX4, a selenoprotein essential for ferroptosis defense, requires selenium acquisition and utilization as the rate-limiting step in its biosynthesis. The selenium metabolic pathway involves the reduction of selenocystine (the oxidized dimer of selenocysteine) to selenocysteine, which is subsequently processed by selenocysteine lyase (SCLY) to generate selenide. This intermediate is then converted to selenophosphate (H_2_SePO_3_^-^) by selenophosphate synthetase 2 (SEPHS2), ultimately facilitating the synthesis of Sec-tRNA^[Ser]Sec^ and GPX4 (Fig. 2a). Our experimental data demonstrate that selenocystine supplementation significantly increases GPX4 protein levels under various conditions, including with or without cystine and RSL3 treatment (Fig. S1a–c). Consistent with these findings, selenocystine effectively suppresses RSL3-induced or cystine deprivation-induced lipid ROS accumulation and cell death in both HT1080 and OS-RC-2 cells (Fig. S1d–g). Analogous to the methionine-to-cysteine conversion pathway, selenomethionine can theoretically be metabolized to selenocysteine through the transsulfuration pathway (Fig. 2a). Notably, although SCLY is the key enzyme responsible for selenocysteine decomposition, emerging evidence suggests that peroxiredoxin 6 (PRDX6) enables selenium-containing compounds to bypass SCLY [26]. thereby supplying selenium for selenoproteins biosynthesis. In this study, we generated a stable SCLY-knockdown HepG2 cell line using sgRNA targeting SCLY (Fig. S1h) and established an RSL3-induced ferroptosis model. Intriguingly, we observed that the anti-ferroptosis effect of selenomethionine was independent of SCLY (Fig. S1i, j). Furthermore, SCLY knockdown did not attenuate the ability of selenomethionine to upregulate GPX4 protein levels (Fig. S1k). To confirm whether selenomethionine can be converted into selenocysteine through the transsulfuration pathway to regulate the protein levels of GPX4, we detected the GPX4 of OS-RC-2, HepG2, and HT1080 cultured in medium with or without selenomethionine, cystine or RSL3. The results showed that selenomethionine supplementation consistently upregulated GPX4 expression (Fig. 2b, c and Fig. S2a). To further elucidate the role of the transsulfuration pathway, we employed DL-Propargylglycine (PAG), a specific inhibitor of cystathionine gamma-lyase (CTH), in HepG2 and HT1080 cells. Surprisingly, PAG treatment can attenuate selenomethionine-mediated GPX4 upregulation (Fig. 2d, e and Fig. S2b), but PAG was unable to block the inhibitory effect selenomethionine on lipid ROS and cell death induced by depriving cystine (Fig. 2f, g). Meanwhile, we generated HepG2 cell lines with stable knockdown of CBS or CTH using specific shRNAs (Fig. 2h). Using these established cell models within an RSL3-induced ferroptosis system, we evaluated the effects of selenomethionine treatment. Notably, our results demonstrated that neither CBS nor CTH knockdown attenuated the anti-ferroptosis activity of selenomethionine (Fig. 2i–j). Based on the similarity in chemical properties between thiol and selenium groups (SH and SeH), we found that 5,5’-Dithiobis-(2-nitrobenzoic acid) (DTNB) could recognize the selenium group of selenocysteine (Fig. 2k). Biochemical assays revealed that while selenocystine effectively restored intracellular thiol and selenol levels, selenomethionine did not (Fig. 2l). Additional experiments confirmed that selenomethionine maintains its ability to upregulate GPX4 and protect against RSL3-induced ferroptosis in H1299, A549, and MEF cell lines (Fig. S2c–i), known to have impaired transsulfuration pathways [27–29]. especially MEF cells, which lack CBS (Fig. S2j, K). Notably, our murine studies revealed that selenomethionine administration retains the capacity to upregulate intracellular GPX4 levels in certain tissues (e.g., heart, stomach, and brain) despite their lack of CBS and CTH expression. (Fig. S2l, m, and n). Taken together, these findings demonstrate that selenomethionine-mediated GPX4 upregulation and ferroptosis protection beyond the transsulfuration pathway.

### The ability of selenomethionine to resist ferroptosis is preserved in GPX4-deficient cells

**Fig. 3:**
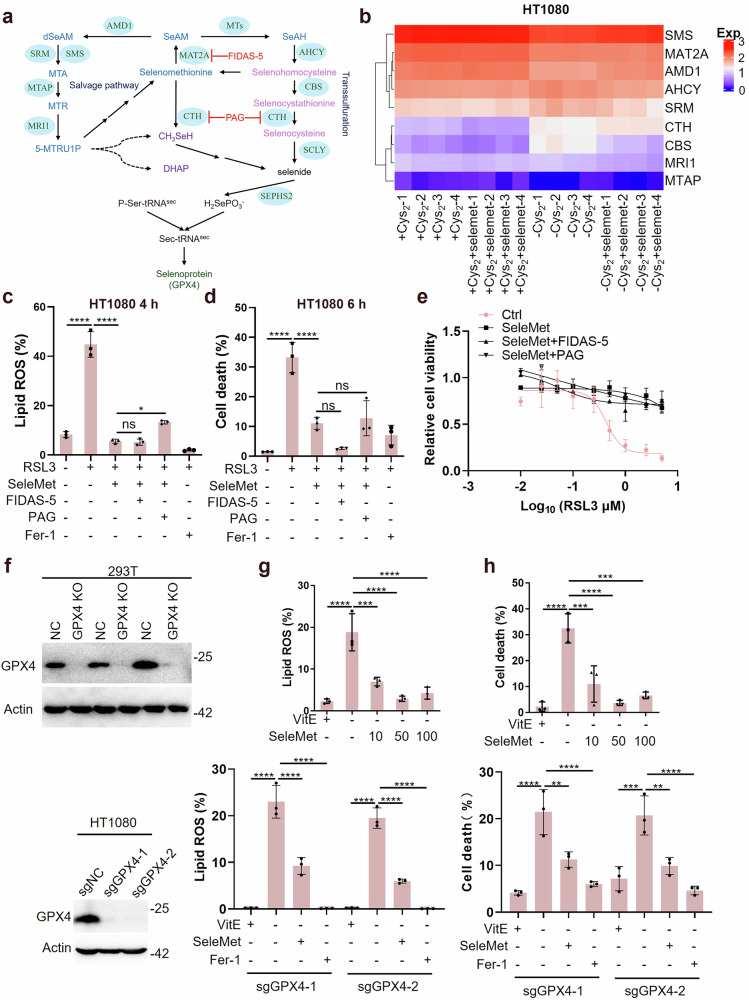
The ability of selenomethionine to resist ferroptosis is preserved in GPX4-deficient cells. **a** Schematic of metabolic processes of selenomethionine (SeleMet). **b** The mRNA levels of the indicated metabolic enzymes in HT1080 were in ± cystine (Cys_2_) medium treated with SeleMet and shown in heatmap. Red color indicated high expression, and blue indicated low expression. **c**, **d** HT1080 cells are cultured medium treated with RSL3, SeleMet, FIDAS-5, PAG, or fer-1 as indicated for 4-6 h (*n* = 3). **c** The lipid ROS levels were determined using flow cytometry (HT1080 for 4 h) (*n* = 3). **d** The cell death levels were quantified using flow cytometry (HT1080 for 6 h) (*n* = 3). **e** The cell viability of HT1080 cells that were treated with RSL3, SeleMet, FIDAS-5, PAG, or fer-1 for 6 h (*n* = 3). **f** The protein levels of the GPX4 in 293 T and HT1080 treated with sgGPX4 were measured by WB. **g**, **h** 293 T and HT1080 KO GPX4 cells are cultured medium treated with Vitamin E (VitE) or SeleMet as indicated for 10–14 h (*n* = 3). **g** The lipid ROS levels were determined using flow cytometry (for 10 h) (*n* = 3). **h** The cell death levels were quantified using flow cytometry (for 14 h) (*n* = 3). Data were represented as mean ± SD with *P* values determined by one-way ANOVA (**c**, **d**, **g**, and **h**). *P < 0.05; **P < 0.01; ***P < 0.001; ****P < 0.0001; ns, nonsignificant.

Our findings suggest that selenomethionine can provide selenium to cells through alternative metabolic pathways beyond the canonical transsulfuration pathway. Previous reports have proposed that selenomethionine may be metabolized through either the methionine salvage pathway or the methionine cleavage pathway (Fig. 3a). These pathways involve key enzymes including S-methyl-5’-thioadenosine phosphorylase (MTAP), methylthioribose-1-phosphate isomerase (MRI1), cystathionine beta-synthase (CBS), and cystathionine gamma-lyase (CTH) (Fig. 3a). Analysis of the Non-Human Primate Cell Atlas (NHPCA) database revealed tissue-specific expression patterns of methionine catabolic enzymes in primates (Fig. S3a). In HT1080 cells, we observed relatively low expression levels of key methionine catabolic enzymes (MTAP, CBS, and CTH) compared to other metabolic enzymes (Fig. 3b), suggesting limited capacity of both the methionine salvage and transsulfuration pathways to process selenomethionine. Interestingly, CTH expression was significantly upregulated in response to both cystine deprivation and selenomethionine supplementation, indicating its potential role in methionine decomposition (Fig. 3b). Consistent with our earlier findings (Fig. 2), while CTH inhibition attenuated selenomethionine-mediated GPX4 upregulation, it did not block selenomethionine’s anti-ferroptosis effects. Furthermore, pharmacological inhibition of MAT2A using FIDAS-5 failed to abrogate selenomethionine’s protective effects against RSL3-induced ferroptosis in HT1080, OS-RC-2, and HepG2 cells (Fig. 3c–e and Fig. S3b–c). These results strongly suggest that selenomethionine’s regulation of GPX4 is not essential for its ferroptosis-inhibitory function. To further validate this conclusion, we generated GPX4-knockout 293 T and HT1080 cells using CRISPR-Cas9 technology (Fig. 3f). Remarkably, selenomethionine maintained its ability to suppress lipid ROS accumulation and cell death in GPX4-deficient cells (Fig. 3g, h). Although selenomethionine exhibits anti-ferroptosis effects independently of GPX4, we hypothesized that it might also function by incorporating into other proteins, such as membrane proteins. To test this hypothesis, we inhibited protein synthesis using cycloheximide (CHX) in HT1080 and HepG2 cells and assessed the impact of selenomethionine on RSL3-induced ferroptosis. Our results demonstrated that CHX partially attenuated the anti-ferroptosis effect of selenomethionine (Fig. S3d, e), whereas it had no influence on the protective activity of ferrostatin-1 (Fig. S3f, g). Additionally, CHX completely abolished selenomethionine-induced GPX4 upregulation (Fig. S3h). These findings suggest that selenomethionine’s anti-ferroptosis activity may, in part, depend on its incorporation into newly synthesized proteins. However, since CHX only partially reduces its protective effect, this mechanism is not strictly required, indicating the existence of additional, protein synthesis-independent pathways. These findings collectively demonstrate that selenomethionine can inhibit ferroptosis through mechanisms independent of GPX4 regulation.

### Selenomethionine acts through reduction capacity to protect against ferroptosis

**Fig. 4:**
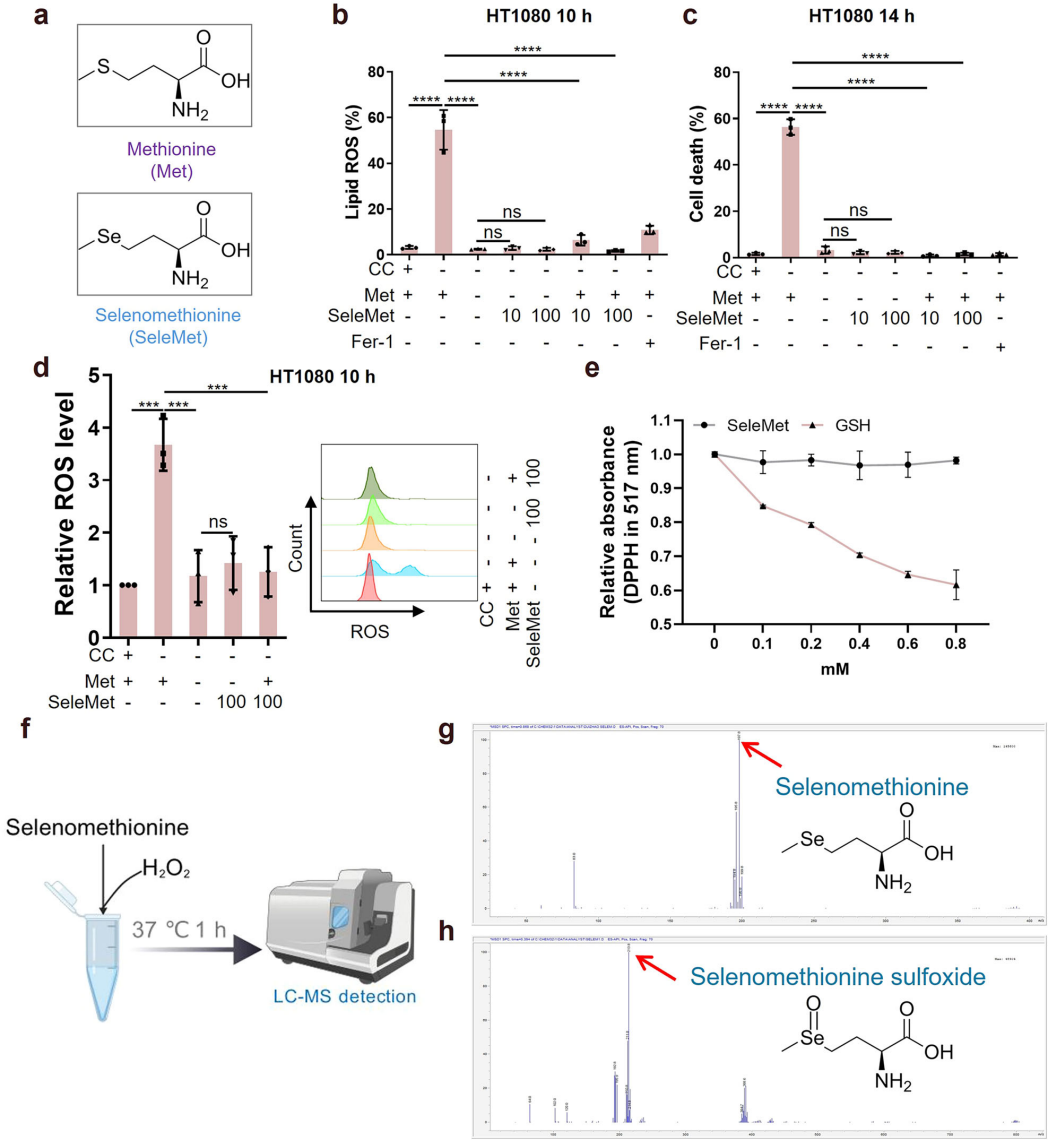
Selenomethionine acts through reduction capacity to protect against ferroptosis. **a** The structural formula of methionine (Met) and selenomethionine (SeleMet). **b**, **c** HT1080 cells are cultured ± cystine (CC) medium treated with met, seleMet, or fer-1 as indicated for 10-14 h (*n* = 3). **b** The lipid ROS levels were determined using flow cytometry (HT1080 for 10 h) (*n* = 3). **c** The cell death levels were quantified using flow cytometry (HT1080 for 14 h) (*n* = 3). **d** HT1080 cells are cultured ± cystine (CC) medium treated with met, seleMet, or fer-1 as indicated for 10 h, then the ROS levels were determined using flow cytometry (*n* = 3). **e** Analysis of the reducibility of GSH and seleMet using 2,2-Diphenyl-1-picrylhydrazyl (DPPH), which has a single electron and can accept one electron or hydrogen ion, with maximum absorption at a wavelength of 517 nm (*n* = 3). **f** The flowchart of chemical reaction and identification. **g** Mass spectrometry detection results of selenometionine and selenomethionine sulfoxide. Data were represented as mean ± SD with *P* values determined by one-way ANOVA (**b**–**d**). ***P < 0.001; ****P < 0.0001; ns, nonsignificant.

While our previous studies established that methionine is essential for ferroptosis execution through S-adenosylmethionine (SAM)-mediated maintenance of intracellular ROS levels [30]. The current investigation reveals that selenomethionine, differing from methionine by only a single atomic substitution, exhibits potent anti-ferroptosis activity (Fig. 4a). Intriguingly, we observed that selenomethionine supplementation does not interfere with methionine deprivation-mediated suppression of cystine deprivation-induced lipid ROS and cell death, and conversely, methionine supplementation does not affect selenomethionine’s anti-ferroptosis effects (Fig. 4b, c). Notably, methionine deprivation or selenomethionine supplementation effectively suppressed ROS accumulation induced by cystine deprivation (Fig. 4d and Fig. S4a). Furthermore, selenomethionine demonstrated significant inhibitory effects on intracellular Fe^2+^ accumulation (Fig. S4b). The O-S-Se (oxygen-sulfur-selenium) redox axis represents a fundamental system for maintaining cellular redox homeostasis. We hypothesized that selenomethionine might directly participate in ROS scavenging. To test this, we employed the 2,2-Diphenyl-1-picrylhydrazyl (DPPH) assay, which measures antioxidant activity through hydrogen atom transfer to the stable nitrogen-centered DPPH radical. The results showed that selenomethionine’s radical scavenging activity operates independently of hydrogen donation (Fig. 4e). To precisely characterize selenomethionine’s ROS-scavenging mechanism, we developed a liquid chromatography-mass spectrometry (LC/MS) method to identify reaction products between selenomethionine and hydrogen peroxide (H_2_O_2_) (Fig. 4f). LC/MS analysis identified selenomethionine sulfoxide as the primary oxidation product (Fig. S4c, d and Fig. 4g, h). Remarkably, supplementation with chemically synthesized selenomethionine sulfoxide to cultured HT1080 cells exerted anti-ferroptosis activity comparable to that of selenomethionine itself (Fig. S5a–d). This suggests the existence of an intracellular reduction system capable of regenerating selenomethionine from its sulfoxide form. Collectively, these findings demonstrate that selenomethionine protects against ferroptosis through a unique redox cycling mechanism involving direct ROS scavenging and subsequent enzymatic reduction of its oxidized form.

### Selenomethionine alleviated cisplatin-induced acute kidney injury

**Fig. 5:**
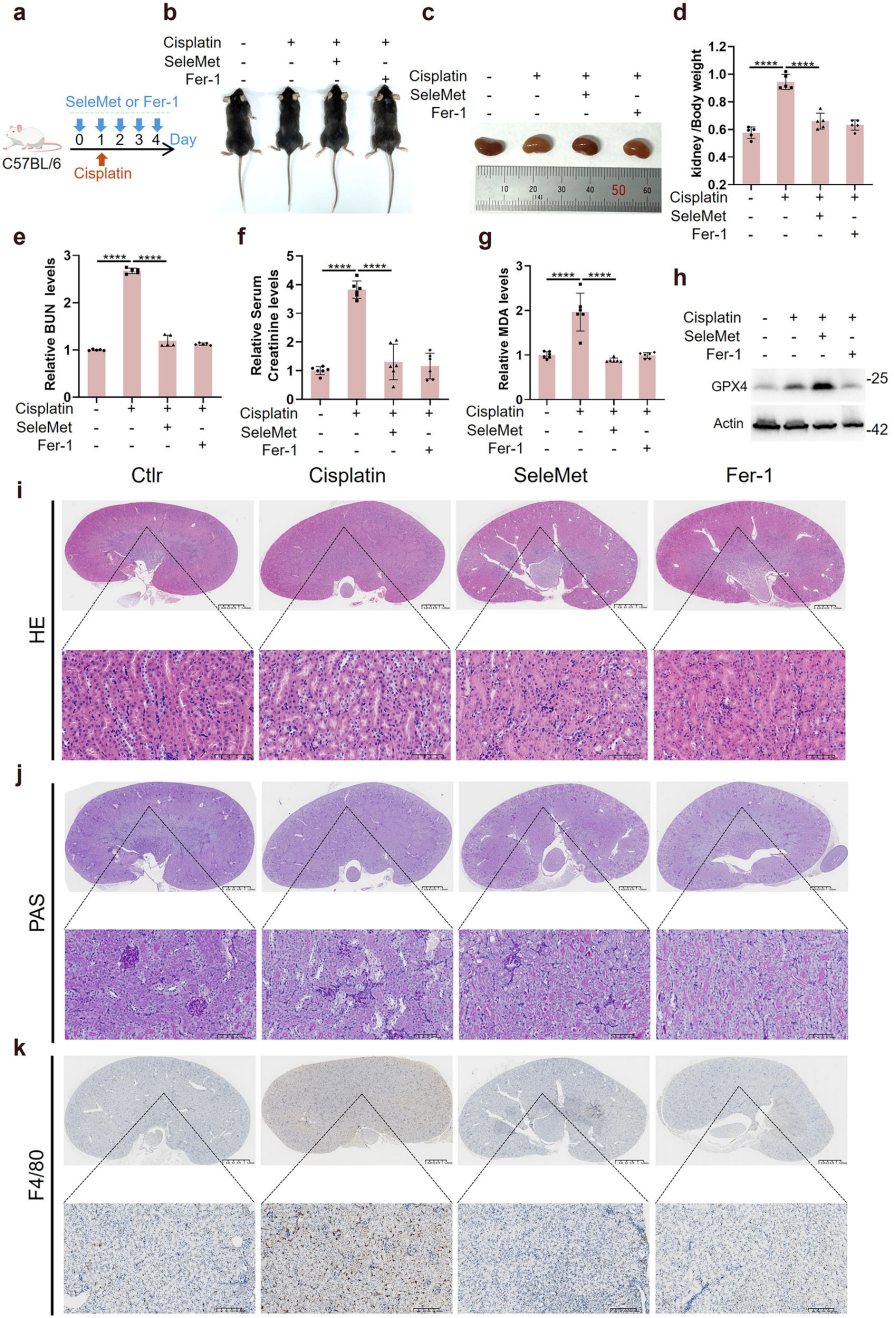
Selenomethionine alleviated cisplatin-induced acute kidney injury. **a** The mice were injected with cisplatin (20 mg/kg) only or combined with selenomethionine (3 mg/kg) or Fer-1 (1 mg/kg) as indicated for 4 days. **b** Morphology of mice after drug treatment. **c** Morphology of kidneys after drug treatment. **d** Kidney/body weight ratio after drug treatment. **e** The blood urea nitrogen (BUN) of mice after drug treatment. **f** The serum creatinine of mice after drug treatment. **g** The MDA of kidney after drug treatment. **h** WB detection of GPX4 protein levels in mice kidneys after drug treatment. **i** HE staining of kidney after drug treatment. **j** PAS staining of kidney after drug treatment. **k** F4/80 staining of kidney after drug treatment. Data were represented as mean ± SD with *P* values determined by one-way ANOVA (**d**–**g**). ****P < 0.0001; ns, nonsignificant.

Previous studies have investigated the regulatory mechanisms of ferroptosis in acute kidney injury (AKI) [31, 32]. In the present study, we established an AKI model through intraperitoneal injection of cisplatin to examine whether selenomethionine could mitigate AKI by suppressing ferroptosis (Fig. 5a). Following treatment with cisplatin combined with either selenomethionine or ferrostatin-1 (fer-1), we measured the body and kidney weights of the mice to determine the kidney-to-body weight ratio. The results demonstrated that cisplatin significantly reduced the body weight of the mice, whereas both selenomethionine and fer-1 partially restored the body weight (Fig. 5b). Additionally, cisplatin-induced kidney edema was ameliorated by selenomethionine or fer-1 (Fig. 5c and Fig. S6a). Consistently, the cisplatin-induced increase in the kidney-to-body weight ratio was also attenuated by selenomethionine or fer-1 (Fig. 5d). Moreover, we observed that selenomethionine significantly alleviated cisplatin-induced AKI, as evidenced by reductions in blood urea nitrogen (BUN) and serum creatinine levels (Fig. 5e, f), as well as decreased expression of IL-6 and TNF-α in the kidney (Fig. S6b, c). Furthermore, we assessed ferroptosis markers and found that cisplatin injection markedly elevated malondialdehyde (MDA) levels in the kidney, which were normalized by selenomethionine or fer-1 (Fig. 5g). Concurrently, selenomethionine significantly upregulated the protein levels of GPX4 in the kidney (Fig. 5h). Finally, histological analysis of cisplatin-induced AKI was performed using hematoxylin and eosin (H&E), periodic acid-Schiff (PAS), and F4/80 staining. The results revealed increased cast formation, intraepithelial vacuolar degeneration, and inflammatory infiltration in cisplatin-treated kidneys compared to those treated with selenomethionine or fer-1 (Fig. 5i-k). Together, our data suggest that selenomethionine alleviate cisplatin-induced AKI through inhibiting ferroptosis in vivo.

## Discussion

In this study, we demonstrate that selenomethionine functions as a ferroptosis inhibitor through dual mechanisms. First, it serves as a bioavailable selenium source to sustain GPX4 biosynthesis even under impaired transsulfuration pathway conditions. Second, independent of its role in GPX4 synthesis, selenomethionine exhibits intrinsic antioxidant properties by directly scavenging ROS through redox-active selenium moieties. These dual mechanisms enable selenomethionine to effectively mitigate ferroptosis-associated pathologies, as exemplified by its therapeutic efficacy in cisplatin-induced acute kidney injury.

While organic and inorganic selenium compounds are known to be metabolized to selenide for selenoprotein biosynthesis [33]. The specific metabolic pathway particularly for selenomethionine, a naturally occurring selenium-containing amino acid remain incompletely characterized. Structurally analogous to methionine (differing only by selenium substitution for sulfur), selenomethionine has been presumed to share metabolic pathways with its sulfur counterpart [34]. Our prior work demonstrated tissue-specific functionality of the transsulfuration pathway in methionine-to-cysteine conversion [30]. Notably, multiple studies have reported limited transsulfuration pathway activity in various cellular systems [27–30], theoretically precluding selenomethionine-derived selenium utilization due to impaired conversion to selenocysteine. Contrary to these expectations, we observed that selenomethionine effectively provides selenium for GPX4 synthesis in H1299, A549, and MEF cell lines with deficient transsulfuration pathways. Our findings reveal three distinct metabolic routes enabling selenium mobilization from selenomethionine: (1) the transsulfuration pathway, (2) methionine salvage pathway, and (3) CTH-mediated methionine degradation. Of particular significance, CTH demonstrates dual functionality not only collaborating with CBS in canonical transsulfuration pathway, but also independently catalyzing methionine cleavage. This mechanistic insight explains our experimental observations: selenomethionine enhances GPX4 protein levels even under transsulfuration-compromised conditions, an effect abrogated by PAG, a selective CTH inhibitor.

Beyond its role in enhancing GPX4 expression, selenomethionine demonstrates non-enzymatic antioxidant activity through direct chemical interactions with ROS to combat ferroptosis. Emerging evidence suggests that hydrogen selenide and selenium species can mitigate ferroptosis through mitochondrial mechanisms involving sulfide quinone oxidoreductase (SQOR)-mediated reduction of ubiquinone [35]. As the primary cellular selenium donor, selenomethionine may undergo metabolic conversion to generate both hydrogen selenide and selenium. These bioactive metabolites could then participate in ubiquinone reduction, thereby establishing a GPX4-independent pathway for ferroptosis suppression. In this investigation, we identified selenomethionine sulfoxide as the primary oxidation product of selenomethionine. Remarkably, exogenous supplementation of selenomethionine sulfoxide retained the capacity to suppress cystine deprivation-induced ferroptosis and ROS accumulation. This observation implies the existence of a cellular reduction system capable of regenerating selenomethionine from its oxidized form, analogous to the GSH-GSSG redox cycle. Notably, methionine-R-sulfoxide reductase B1 (MSRB1), a stereospecific enzyme that catalyzes the reduction of methionine-sulfoxide to methionine emerges as a prime candidate for mediating this redox cycling [36]. These findings underscore the critical need to establish whether MSRB1 or related enzymatic machinery participates in the reduction of selenomethionine sulfoxide, as this mechanistic insight is essential for fully elucidating the antioxidant mechanisms underpinning selenomethionine’s anti-ferroptosis activity.

In summary, our study establishes selenomethionine as a novel dual-mechanism inhibitor of ferroptosis. Specifically, selenomethionine confers ferroptosis protection through two distinct pathways : (1) a GPX4-dependent mechanism involving selenium provision via multiple metabolic routes to sustain GPX4 biosynthesis, and (2) a GPX4-independent mechanism mediated through direct ROS scavenging via its intrinsic redox-active properties. Furthermore, we demonstrate the therapeutic relevance of these dual functions by showing selenomethionine’s efficacy in alleviating cisplatin-induced acute kidney injury. This multimodal action, combining enzymatic support with direct antioxidant activity, positions selenomethionine as a promising therapeutic candidate for ferroptosis-associated pathologies.

## Methods

### Cell culture

HT1080 cells, OS-RC-2, HepG2, H1299, A549, and mouse embryonic fibroblast (MEF) cells were obtained from the American Tissue Culture Collection (ATCC) and authenticated by the distributors. All cell lines were confirmed to be free of mycoplasma contamination. HepG2, HT1080, and MEF cells were cultured in Dulbecco’s modified Eagle’s medium (DMEM) supplemented with 10% fetal bovine serum (FBS) and 1% penicillin-streptomycin (P/S) at 37 °C with 5% CO_2_. OS-RC-2, A549, and H1299 cells were cultured in RPMI-1640 medium supplemented with 10% FBS and 1% P/S at 37 °C with 5% CO_2_.

### Antibodies and reagents

Primary antibodies used were anti-GPX4 (1:1000 for WB, A11243, Abclonal), anti-CBS (1:1000 for WB, A11612, Abclonal), anti-CTH (1:1000 for WB, A6121, Abclonal), anti-SCLY (1:1000 for WB, A15842, Abclonal), anti-GAPDH (1:1000 for WB, A19056, Abclonal), and anti-Actin (1:1000 for WB, HC201-01, TransGen). The secondary antibodies included HRP-conjugated Goat anti-Mouse IgG (H + L) (1:5000 for WB, AS003, Abclonal) and HRP-conjugated Goat anti-Rabbit IgG (H + L) (1:5000 for WB, AS014, Abclonal). The compounds used are Ferrostain-1 (Fer-1) (S7243, Selleck), Propidium iodide (PI) (P4170, Sigma), BODIPY581/591 C11 (D3861, ThermoFisher), DCFH2-DA (D6883, Sigma), Cystine (30200, Sigma), Methionine (Met) (M9625, Sigma), RhoNox-1 (HY-D1533, MCE), α-Vitamin E ((+)-α-Tocopherol) (HY-N0683, MCE), RSL3 (HY-100218A, MCE), DL-Propargylglycine (HY-W040124, MCE), Selenomethionine (HY-B1000, MCE), L-Selenocystine (HY-129960, MCE), Cisplatin (HY-17394, MCE), Cycloheximide (HY-12320, MCE), FDA-Approved Drug Library (HY-L022, MCE), Natural Product Library (HY-L021, MCE), Dulbecco’s Modified Eagle’s Medium high glucose without L-methionine, L-cystine, and L-glutamine (D0422-100 ml, Sigma), and Penicillin-Streptomycin (P06-07100, Pan Biotech). Other reagents were obtained from MCE or Sigma.

### Small molecule library screening

The small molecule library containing 6276 compounds was purchased from MCE (FDA-Approved Drug Library (HY-L022) and Natural Product Library (HY-L021). HT1080 cells were plated in 96-well plates at a density of 10000 cells per well. After 12 h, the cells were treated with indicated compounds (10 μM) or DMSO (10 μM) and RSL3 (0.2 μM) for 24 h. Cell was assessed using propidium iodide (2 μg/mL) and Hoechst (1 μg/mL), and the data were subsequently collected by CX7 LED Pro.

### Cell viability assay

Cell viability was determined using the Cell Counting Kit-8 (CCK-8; HY-K0301, MCE). Briefly, cells were seeded in 96-well plates at a density of 20,000 cells per well and incubated for 10 h. Following experimental treatments, 10 μL of CCK-8 reagent was added to each well containing 100 μL of culture medium. Cells were then incubated with the reagent for 1 h at 37 °C under 5% CO_2_. Absorbance was measured at 450 nm using a microplate reader (Thermo Scientific).

### Measurement of Fe^2+^ by RhoNox-1

Cells were seeded in 24-well plates at a density of 5 × 10⁴ cells per well and cultured in fresh medium for 12 h. After experimental treatments, 10 μM RhoNox-1 (a selective Fe²⁺ fluorescent probe) was added to the culture medium and incubated with cells for 25 min at 37 °C. Cells were subsequently trypsinized, washed twice with PBS, and resuspended in 0.3 mL of PBS containing 5% fetal bovine serum (FBS) for analysis. Flow cytometry analysis was carried out with Attune NxT Acoustic Focusing Cytometer (Thermo Fisher Scientific), and acquired data were analyzed using FlowJo (version 10).

### Measurement of lipid ROS by BODIPY-C11

The experiment was carried out as described previously [37]. Briefly, cells were seeded in 24-well plates at a density of 5 × 10⁴ cells per well and cultured in fresh medium for 12 h. Following experimental treatments, 4 μM BODIPY 581/591 C11 (a lipid peroxidation-sensitive fluorescent probe) was added to the culture medium and incubated with cells for 25 min at 37 °C. Cells were then trypsinized, washed twice with phosphate-buffered saline (PBS), and resuspended in 0.3 mL of PBS containing 5% fetal bovine serum (FBS) for flow cytometry analysis. Cells positive for BODIPY 581/591 C11 were defined as lipid ROS accumulation. Flow cytometry analysis was carried out with Attune NxT Acoustic Focusing Cytometer (Thermo Fisher Scientific), and acquired data were analyzed using FlowJo (version 10).

### Measurement of cell death by PI

The experiment was carried out as described previously [37]. Briefly, cells were seeded in 24-well plates at a density of 5 × 10⁴ cells per well and cultured in fresh medium for 12 h. Following the indicated treatment, 1 μg/mL propidium iodide (PI) was added to the cell culture medium and incubated for 30 min. Then cells were trypsinized, washed and resuspended in 0.3 mL of PBS containing 5% fetal bovine serum for flow cytometry analysis of the cell death level. Flow cytometry analysis was carried out with Attune NxT Acoustic Focusing Cytometer (Thermo Fisher Scientific), and acquired data were analyzed using FlowJo (version 10).

### Measurement of ROS by DCFH2-DA

Cells were seeded in 24-well plates at a density of 5 × 10⁴ cells per well and cultured in fresh medium for 12 h. Following the indicated treatment, 10 μM DCFH2-DA was added to the cell culture medium and incubated for 25 min. Cells were then trypsinized, washed and resuspended in 0.3 mL of PBS containing 5% fetal bovine serum for flow cytometry analysis of the cellular ROS levels. Flow cytometry analysis was carried out with Attune NxT Acoustic Focusing Cytometer (Thermo Fisher Scientific), and acquired data were analyzed using FlowJo (version 10).

### Quantitative real-time PCR

Total RNA was extracted from cells using TRNzol (TIANGEN, China). Total RNA was then reverse transcribed and real-time PCR with HiScript IV All-in-One Ultra RT SuperMix for qPCR (R433-01, Vazyme) was performed on the qTOWER³ (analytikjena) real-time PCR instrument. The mRNA expression of genes was normalized to the expression of *β-actin* gene. Data was analyzed using the comparative cycling threshold method. The sequences of the primers used for Quantitative real-time PCR were:

GPX4, forward CGGAATTCATGAGCCTCGGCCGCCTTTG, reverse CCGCTCGAGGAAATA- GTGGGGCAGGTCCT; CBS, forward CAGAGGATAAGGAAGCCAAG, reverse TCCCAATCT TGTTGATTCTGAC; CTH, forward CATGAGTTGGTGAAGCGTCAG, reverse AGCTCTCGG- CCAGAGTAAATA; β-actin, forward GTTGTCGACGACGAGCG, reverse GCACAGAGCCTC- GCCTT. IL-6 β, forward CTGCAAGAGACTTCCATCCAG, reverse AGTGGTATAGACAGGT- CTGTTGG; TNF-α forward GACCCTCACACTCAGATCATCTTCT, reverse TCCTCCACTTG- GTGGTTTGC.

### Western blot

Cells were lysed in cell lysis buffer (P0013, Beyotime) for 30 min and subjected to SDS-PAGE for western blot. Membranes were blocked with 5% (wt/vol) nonfat dried milk in TBS containing 0.1% Tween 20 (TBST) and subsequently incubated with the appropriate antibodies in 5% (wt/vol) nonfat dried milk in TBST overnight at 4 °C. All primary antibody incubations (1:1000 in TBST, overnight at 4 °C) were followed by incubation with a secondary HRP-conjugated antibody (1:5000 in TBST, 1 h at room temperature), and the signals were visualized using a chemiluminescent HRP substrate on a chemiluminescence imaging system (CHAMPCHEMI^TM^ Chemiluminescent Imaging and Analysis System). Full and uncropped western blot originals have been uploaded as Supplemental Material.

### CRISPR/Cas9-mediated gene knockout

CRISPR/Cas9 technology was employed to knock out target genes in the cell lines. sgRNAs were cloned into the lentiCRISPR-v2-puro (addgene Cat#98290). The following sgRNA sequences were used: sgGPX4-1, 5’- CACCGTTTTCCGCCAAGGACATCGA -3’; sgGPX4-2, 5’- CACCGCACGCCCGATACGCTGAGTG -3’; sgSCLY, 5’- CUAUAAUGCAACGACUCCCC-3’. Then, the edited vectors were packaged into lentiviruses in HEK-293T cells with the psPAX2 and pMD2.G-VSV-G plasmids. The virus-containing medium was harvested after 48 h, after which cells were infected with virus and 1 µg/mL polybrene. Puromycin (1 µg/mL) was used for selection. CRISPR/Cas9 knockout efficiency was confirmed by immunoblot analysis.

### shRNA-mediated gene knockdown

shRNAs were cloned into the pLKO.1 puro (addgene Cat#8453). The following sgRNA sequences were used:

shCBS-F, 5’-CCGGGCGGAACTACATGACCAAGTTCTCGAGAACTTGGTCATGTAGTTCC GCTTTTTG-3’; shCBS-R, 5’-AATTCAAAAAGCGGAACTACATGACCAAGTTCTCGAGAA CTTGGTCATGTAGTTCCGC-3’; shCTH-F, 5’- CCGGCCTTCATAATAGACTTCGTTTCTCGAGAAACGAAGTCTATTATGAAG GTTTTTG-3’; shCTH-R, 5’- AATTCAAAAACCTTCATAATAGACTTCGTTTCTCGAGAAAC GAAGTCTATTATGAAGG-3’.

Then, the edited vectors were packaged into lentiviruses in HEK-293T cells with the psPAX2 and pMD2.G-VSV-G plasmids. The virus-containing medium was harvested after 48 h, after which cells were infected with virus and 1 µg/mL polybrene. Puromycin (1 µg/mL) was used for selection. knockout efficiency was confirmed by immunoblot analysis.

### Cisplatin induced acute kidney injury

In this study, we established a cisplatin-induced renal injury model in mice (C57BL/6, Male, 8 weeks old). The dosing regimen was as follows: cisplatin (20 mg/kg) was administered via single intraperitoneal injection on day 2, while selenomethionine (3 mg/kg) and ferrostatin-1 (1 mg/kg) were administered daily by intraperitoneal injection for four consecutive days. All mice being humanely euthanized 24 h after the final administration. This study was conducted in strict compliance with the ethical guidelines approved by the Animal Ethics Committee of Zhejiang Sci-Tech University (Approval No. 202501017), ensuring full adherence to animal welfare regulations.

### Histology

The kidneys of mouse were fixed in 4% paraformaldehyde for 24 h, followed by dehydration, embedding in paraffin, and serial sectioning at a thickness of 4 µm. The sections were then stained for hematoxylin and eosin (H&E), periodic acid-Schiff (PAS), and F4/80. Microscopic imaging of the H&E, PAS, and F4/80 staining. Sections were performed using a Zeiss Axio Vert.A1.

### Statistical analysis

All experiments were performed with a minimum of three independent replications. Statistical analysis was carried out with GraphPad Prism 10.4.0. All data are shown as mean ± SD. One-way analysis of variance (ANOVA) followed by Tukey’s post-hoc test was utilized for multiple group comparison. In all tests, *P* values of less than 0.05 were considered statistically significant.

## Supplementary information

Supplementary figure legends

Supplementary figure S1

Supplementary figure S2

Supplementary figure S3

Supplementary figure S4

Supplementary figure S5

Supplementary figure S6

## References

[1] Stockwell BR. Ferroptosis turns 10: Emerging mechanisms, physiological functions, and therapeutic applications. Cell. 2022;185:2401–21. 35803244 10.1016/j.cell.2022.06.003 PMC9273022

[2] Dixon SJ, Lemberg KM, Lamprecht MR, Skouta R, Zaitsev EM, Gleason CE, et al. Ferroptosis: an iron-dependent form of nonapoptotic cell death. Cell. 2012;149:1060–72. 22632970 10.1016/j.cell.2012.03.042 PMC3367386

[3] Xia CY, Gao LC, Min JS, Fu CY. Mitochondria ATP pro-ferroptosis by adjusting the conversion of PUFA to PUFA-PLs. Medcomm-Oncol. 2025;4:e70038.

[4] Sassano ML, Tyurina YY, Diokmetzidou A, Vervoort E, Tyurin VA, More S, et al. Endoplasmic reticulum-mitochondria contacts are prime hotspots of phospholipid peroxidation driving ferroptosis. Nat Cell Biol. 2025;27:902–17. 40514428 10.1038/s41556-025-01668-z PMC12173944

[5] Green DR. The coming decade of cell death research: five riddles. Cell. 2019;177:1094–107. 31100266 10.1016/j.cell.2019.04.024 PMC6534278

[6] Newton K, Strasser A, Kayagaki N, Dixit VM. Cell death. Cell. 2024;187:235–56. 38242081 10.1016/j.cell.2023.11.044

[7] Tsvetkov P, Coy S, Petrova B, Dreishpoon M, Verma A, Abdusamad M, et al. Copper induces cell death by targeting lipoylated TCA cycle proteins. Science. 2022;375:1254–61. 35298263 10.1126/science.abf0529 PMC9273333

[8] Co HKC, Wu CC, Lee YC, Chen SH. Emergence of large-scale cell death through ferroptotic trigger waves. Nature. 2024;631:654–62. 38987590 10.1038/s41586-024-07623-6 PMC11639682

[9] Jiang X, Stockwell BR, Conrad M. Ferroptosis: mechanisms, biology and role in disease. Nat Rev Mol Cell Biol. 2021;22:266–82. 33495651 10.1038/s41580-020-00324-8 PMC8142022

[10] Stockwell BR, Friedmann Angeli JP, Bayir H, Bush AI, Conrad M, Dixon SJ, et al. Ferroptosis: a regulated cell death nexus linking metabolism, redox biology, and disease. Cell. 2017;171:273–85. 28985560 10.1016/j.cell.2017.09.021 PMC5685180

[11] Conrad M, Pratt DA. The chemical basis of ferroptosis. Nat Chem Biol. 2019;15:1137–47. 31740834 10.1038/s41589-019-0408-1

[12] Yang WS, SriRamaratnam R, Welsch ME, Shimada K, Skouta R, Viswanathan VS, et al. Regulation of ferroptotic cancer cell death by GPX4. Cell. 2014;156:317–31. 24439385 10.1016/j.cell.2013.12.010 PMC4076414

[13] Yao Y, Chen Z, Zhang H, Chen C, Zeng M, Yunis J, et al. Selenium-GPX4 axis protects follicular helper T cells from ferroptosis. Nat Immunol. 2021;22:1127–39. 34413521 10.1038/s41590-021-00996-0

[14] Mayr L, Grabherr F, Schwarzler J, Reitmeier I, Sommer F, Gehmacher T, et al. Dietary lipids fuel GPX4-restricted enteritis resembling Crohn’s disease. Nat Commun. 2020;11:1775. 32286299 10.1038/s41467-020-15646-6 PMC7156516

[15] Friedmann Angeli JP, Schneider M, Proneth B, Tyurina YY, Tyurin VA, Hammond VJ, et al. Inactivation of the ferroptosis regulator Gpx4 triggers acute renal failure in mice. Nat Cell Biol. 2014;16:1180–91. 25402683 10.1038/ncb3064 PMC4894846

[16] Bersuker K, Hendricks JM, Li Z, Magtanong L, Ford B, Tang PH, et al. The CoQ oxidoreductase FSP1 acts parallel to GPX4 to inhibit ferroptosis. Nature. 2019;575:688–92. 31634900 10.1038/s41586-019-1705-2 PMC6883167

[17] Doll S, Freitas FP, Shah R, Aldrovandi M, da Silva MC, Ingold I, et al. FSP1 is a glutathione-independent ferroptosis suppressor. Nature. 2019;575:693–8. 31634899 10.1038/s41586-019-1707-0

[18] Tonnus W, Maremonti F, Gavali S, Schlecht MN, Gembardt F, Belavgeni A, et al. Multiple oestradiol functions inhibit ferroptosis and acute kidney injury. Nature. 2025;645:1011–9. 40804518 10.1038/s41586-025-09389-x PMC12460175

[19] Kraft VAN, Bezjian CT, Pfeiffer S, Ringelstetter L, Muller C, Zandkarimi F, et al. GTP Cyclohydrolase 1/Tetrahydrobiopterin Counteract Ferroptosis through Lipid Remodeling. ACS Cent Sci. 2020;6:41–53. 31989025 10.1021/acscentsci.9b01063 PMC6978838

[20] Mao C, Liu X, Zhang Y, Lei G, Yan Y, Lee H, et al. DHODH-mediated ferroptosis defence is a targetable vulnerability in cancer. Nature. 2021;593:586–90. 33981038 10.1038/s41586-021-03539-7 PMC8895686

[21] Freitas FP, Alborzinia H, Dos Santos AF, Nepachalovich P, Pedrera L, Zilka O, et al. 7-Dehydrocholesterol is an endogenous suppressor of ferroptosis. Nature. 2024;626:401–10. 38297129 10.1038/s41586-023-06878-9

[22] Li Y, Ran Q, Duan Q, Jin J, Wang Y, Yu L, et al. 7-Dehydrocholesterol dictates ferroptosis sensitivity. Nature. 2024;626:411–8. 38297130 10.1038/s41586-023-06983-9 PMC11298758

[23] Mishima E, Ito J, Wu Z, Nakamura T, Wahida A, Doll S, et al. A non-canonical vitamin K cycle is a potent ferroptosis suppressor. Nature. 2022;608:778–83. 35922516 10.1038/s41586-022-05022-3 PMC9402432

[24] Gaschler MM, Stockwell BR. Lipid peroxidation in cell death. Biochem Biophys Res Commun. 2017;482:419–25. 28212725 10.1016/j.bbrc.2016.10.086 PMC5319403

[25] Labunskyy VM, Hatfield DL, Gladyshev VN. Selenoproteins: molecular pathways and physiological roles. Physiol Rev. 2014;94:739–77. 24987004 10.1152/physrev.00039.2013 PMC4101630

[26] Chen Z, Inague A, Kaushal K, Fazeli G, Schilling D, Xavier da Silva TN, et al. PRDX6 contributes to selenocysteine metabolism and ferroptosis resistance. Mol Cell. 2024;84:46–4559.e9. 10.1016/j.molcel.2024.10.027 39547224

[27] Kang YP, Mockabee-Macias A, Jiang C, Falzone A, Prieto-Farigua N, Stone E, et al. Non-canonical glutamate-cysteine ligase activity protects against ferroptosis. Cell Metab. 2021;33:174–89.e7. 33357455 10.1016/j.cmet.2020.12.007 PMC7839835

[28] Cao J, Chen X, Jiang L, Lu B, Yuan M, Zhu D, et al. DJ-1 suppresses ferroptosis through preserving the activity of S-adenosyl homocysteine hydrolase. Nat Commun. 2020;11:1251. 32144268 10.1038/s41467-020-15109-y PMC7060199

[29] Xia C, Xing X, Zhang W, Wang Y, Jin X, Wang Y, et al. Cysteine and homocysteine can be exploited by GPX4 in ferroptosis inhibition independent of GSH synthesis. Redox Biol. 2024;69:102999. 38150992 10.1016/j.redox.2023.102999 PMC10829872

[30] Xia C, Peng P, Zhang W, Xing X, Jin X, Du J, et al. Methionine-SAM metabolism-dependent ubiquinone synthesis is crucial for ROS accumulation in ferroptosis induction. Nat Commun. 2024;15:8971. 39420002 10.1038/s41467-024-53380-5 PMC11487270

[31] Lin Q, Li S, Jin H, Cai H, Zhu X, Yang Y, et al. Mitophagy alleviates cisplatin-induced renal tubular epithelial cell ferroptosis through ROS/HO-1/GPX4 axis. Int J Biol Sci. 2023;19:1192–210. 36923942 10.7150/ijbs.80775 PMC10008689

[32] Deng Y, Zeng L, Liu H, Zuo A, Zhou J, Yang Y, et al. Silibinin attenuates ferroptosis in acute kidney injury by targeting FTH1. Redox Biol. 2024;77:103360. 39326069 10.1016/j.redox.2024.103360 PMC11462067

[33] Fujita H, Tanaka YK, Ogata S, Suzuki N, Kuno S, Barayeu U, et al. PRDX6 augments selenium utilization to limit iron toxicity and ferroptosis. Nat Struct Mol Biol. 2024;31:1277–85. 38867112 10.1038/s41594-024-01329-z PMC11327102

[34] Okuno T, Ueno H, Nakamuro K. Cystathionine gamma-lyase contributes to selenomethionine detoxification and cytosolic glutathione peroxidase biosynthesis in mouse liver. Biol Trace Elem Res. 2006;109:155–71. 16444005 10.1385/BTER:109:2:155

[35] Lee N, Park SJ, Lange M, Tseyang T, Doshi MB, Kim TY, et al. Selenium reduction of ubiquinone via SQOR suppresses ferroptosis. Nat Metab. 2024;6:343–58. 38351124 10.1038/s42255-024-00974-4 PMC11694790

[36] Le DT, Liang X, Fomenko DE, Raza AS, Chong CK, Carlson BA, et al. Analysis of methionine/selenomethionine oxidation and methionine sulfoxide reductase function using methionine-rich proteins and antibodies against their oxidized forms. Biochemistry. 2008;47:6685–94. 18505275 10.1021/bi800422s PMC2844923

[37] Gao M, Monian P, Quadri N, Ramasamy R, Jiang X. Glutaminolysis and Transferrin Regulate Ferroptosis. Mol Cell. 2015;59:298–308. 26166707 10.1016/j.molcel.2015.06.011 PMC4506736

