# Supplementary material for: Selenomethionine as a dual-mechanism ferroptosis inhibitor: selenium-supply-driven GPX4 biosynthesis beyond transsulfuration and reductive-capacity-mediated ROS scavenging independent of GPX4 activity
Source: Cell Death Dis. 2026 Feb 14;17(1):224. doi: 10.1038/s41419-026-08466-x (PMC12921039; doi:10.1038/s41419-026-08466-x)
Supplement: Supplementary file 1 — Supplementary figure legends [file 41419_2026_8466_MOESM1_ESM.docx]

**F****igure S1. Involvement of selemethionine in GPX4 synthesis and ferroptosis inhibition is independent of transsulfuration, related to Figure 2**

**(a)** The protein levels of the GPX4 in HepG2 cells are cultured in medium treated with selenocystine (SeleCys_2_) were measured by WB. **(b)** The protein levels of the GPX4 in HepG2 cells are cultured in ± CC (cystine) medium treated with SeleCys_2_ were measured by WB. **(c)** The protein levels of the GPX4 in HepG2 and HT1080 cells are cultured in medium treated with RSL3 or SeleCys_2_ were measured by WB. **(d, e)** HT1080 and OS-RC-2 cells are cultured in medium treated with RSL3, SeleCys_2_, or fer-1 as indicated for 4-6 h (*n* = 3). (d) The lipid ROS levels were determined using flow cytometry (for 4 h) (*n* = 3). (e) The cell death levels were quantified using flow cytometry (for 6 h) (*n* = 3). **(f, g)** HT1080 cells are cultured in ± CC (cystine) medium treated with SeleCys_2_ or fer-1 as indicated for 10-14 h (*n* = 3). (f) The lipid ROS levels were determined using flow cytometry (HT1080 for 10 h) (*n* = 3). (g) The cell death levels were quantified using flow cytometry (HT1080 for 14 h) (*n* = 3). **(h)** The protein levels of the SCLY in HepG2 cells treated with sgSCLY were measured by WB. **(i, j)** HepG2 (sgNC and sgSCLY) cells are treated with RSL3 or SeleMet as indicated for 4-6 h (*n* = 3). (i) The lipid ROS levels were determined using flow cytometry (for 4 h) (*n* = 3). (j) The cell death levels were quantified using flow cytometry (for 6 h) (*n* = 3). **(k)** HepG2 (sgNC and sgSCLY) cells are treated with RSL3 or SeleMet as indicated for 4 h, the protein levels of the GPX4 were measured by WB. Data were represented as mean ± SD with *P* values determined by one-way ANOVA (d-g, i, and j). *****P* < 0.0001; ns, nonsignificant.

**Figure S2. Involvement of selenomethionine in GPX4 synthesis and ferroptosis inhibition beyond transsulfuration, related to Figure 2**

**(a)** The protein levels of the GPX4 in OS-RC-2, HepG2, and HT1080 cells are cultured in medium treated with SeleMet were measured by WB. **(b)** The protein levels of the GPX4 in HT1080 cells are cultured in ± CC (cystine) medium treated with SeleMet or DL-Propargylglycine (PAG, 100 μM) were measured by WB. **(c)** The protein levels of the GPX4 in A549 and H1299 cells are cultured in medium treated with RSL3 or SeleMet were measured by WB. **(d, e)** H1299 cells are cultured in medium treated with RSL3, SeleMet, or fer-1 as indicated for 4-6 h (*n* = 3). (d) The lipid ROS levels were determined using flow cytometry (H1299 for 4 h) (*n* = 3). (e) The cell death levels were quantified using flow cytometry (H1299 for 6 h) (*n* = 3). **(f)** The protein levels of the GPX4 in MEF cells are cultured in medium treated with SeleMet were measured by WB. **(g)** The protein levels of the GPX4 in MEF cells are cultured in medium treated with SeleMet or DL-Propargylglycine (PAG, 100 μM) were measured by WB. **(h, i)** MEF cells are cultured in medium treated with RSL3, SeleMet, or fer-1 as indicated for 4-6 h (*n* = 3). (h) The lipid ROS levels were determined using flow cytometry (MEF for 4 h) (*n* = 3). (i) The cell death levels were quantified using flow cytometry (MEF for 6 h) (*n* = 3). **(j)** The expression of the CBS and CTH in MEF, HepG2 and HT1080 cells were measured by qPCR. **(k)** The proteins of CBS and CTH in MEF, HepG2 and HT1080 cells were measured by WB. **(l)** Mouse experimental processing flowchart. **(m)** The proteins of CBS and CTH in mouse treated with seleMet (3 mg/kg), were measured by WB. **(n)** The expression of the CBS and CTH in mouse were measured by qPCR. Data were represented as mean ± SD with *P* values determined by one-way ANOVA (d, e, h, and i). ****P* < 0.001; *****P* < 0.0001; ns, nonsignificant.

**Figure S3. The ability of selenomethionine to resist ferroptosis is preserved in GPX4-deficient cells, related to Figure 3**

**(a)** Global uniform manifold approximation and projection (UMAP) visualization of indicated enzymes expressions across all tissues in the monkey based on the single cell transcriptomics data from Non-Human Primate Cell Atlas (NHPCA) database. Blue color indicated low expression, and the red color indicated high expression. **(b, c)** OS-RC-2 and HepG2 cells are cultured in medium treated with RSL3, SeleMet, FIDAS-5, PAG, or fer-1 as indicated for 4-6 h (*n* = 3). (b) The lipid ROS levels were determined using flow cytometry (for 4 h) (*n* = 3). (c) The cell death levels were quantified using flow cytometry (for 6 h) (*n* = 3). **(d, e)** HT1080 and HepG2 cells are cultured in medium treated with RSL3, SeleMet, fer-1, or Cycloheximide (CHX, 1 μM, 10 μM, or 100 μM) as indicated for 4-6 h (*n* = 3). (d) The lipid ROS levels were determined using flow cytometry (for 4 h) (*n* = 3). (e) The cell death levels were quantified using flow cytometry (for 6 h) (*n* = 3). **(f, g)** HepG2 cells are cultured in medium treated with RSL3, fer-1, or CHX as indicated for 4-6 h (*n* = 3). (f) The lipid ROS levels were determined using flow cytometry (for 4 h) (*n* = 3). (g) The cell death levels were quantified using flow cytometry (for 6 h) (*n* = 3). **(h)** HT1080 and HepG2 cells are cultured in medium treated with RSL3, SeleMet, or CHX as indicated for 4 h, the protein levels of the GPX4 were measured by WB. Data were represented as mean ± SD with *P* values determined by one-way ANOVA (b-g). **P* < 0.05; ***P* < 0.01; ****P* < 0.001; *****P* < 0.0001; ns, nonsignificant.

**Figure S4. Selenomethionine acts through reduction capacity to protect against ferroptosis, related to Figure 4**

**(a)** HT1080 cells are cultured ± cystine (CC) medium treated with seleMet as indicated for 10 h, then the ROS levels were determined using flow cytometry (*n* = 3). **(b)** HT1080 cells are cultured ± cystine (CC) medium treated with seleMet as indicated for 10 h, then the Fe^2+^ levels were determined using flow cytometry (*n* = 3). **(c, d)** Liquid Chromatograph detection results of selenometionine (c) and selenomethionine sulfoxide (d). Data were represented as mean ± SD with *P* values determined by one-way ANOVA (a and b). **P* < 0.05; ***P* < 0.01; ****P* < 0.001; ns, nonsignificant.

**Figure S5. Selenomethionine acts through reduction capacity to protect against ferroptosis,** **related to Figure 4**

**(a)** Chemical synthesis route of Selenomethionine sulfoxide and LC-MS detection of key intermediate products. **(b, c)** HT1080 cells are cultured in ± CC (Cystine) medium treated with SeleMet or SeleMet sulfoxide as indicated for 10-14 h (*n* = 3). (b) The lipid ROS levels were determined using flow cytometry (HT1080 for 10 h) (*n* = 3). (c) The cell death levels were quantified using flow cytometry (HT1080 for 14 h) (*n* = 3). **(d)** The cell viability of HT1080 cells that were treated with RSL3, SeleMet (100 μM), or SeleMet sulfoxide for 6 h (*n* = 3). Data were represented as mean ± SD with *P* values determined by one-way ANOVA (b and c). *****P* < 0.0001; ns, nonsignificant.

**Figure S6. Selenomethionine alleviated cisplatin-induced acute kidney injury,** **related to Figure 5**

**(a)** The mice were injected with cisplatin (20 mg/kg) only or combined with selenomethionine (3 mg/kg) or Fer-1 (1 mg/kg) as indicated for 4 days, kidney weight ratio after drug treatment. **(b)** The expression of IL-6 β at kidney after drug treatment. **(c)** The expression of TNF-α at kidney after drug treatment. Data were represented as mean ± SD with *P* values determined by one-way ANOVA (a-c). ****P* < 0.001; *****P* < 0.0001; ns, nonsignificant.
