# Supplementary figures and images for: Selenomethionine as a dual-mechanism ferroptosis inhibitor: selenium-supply-driven GPX4 biosynthesis beyond transsulfuration and reductive-capacity-mediated ROS scavenging independent of GPX4 activity
Source: Cell Death Dis. 2026 Feb 14;17(1):224. doi: 10.1038/s41419-026-08466-x (PMC12921039; doi:10.1038/s41419-026-08466-x)

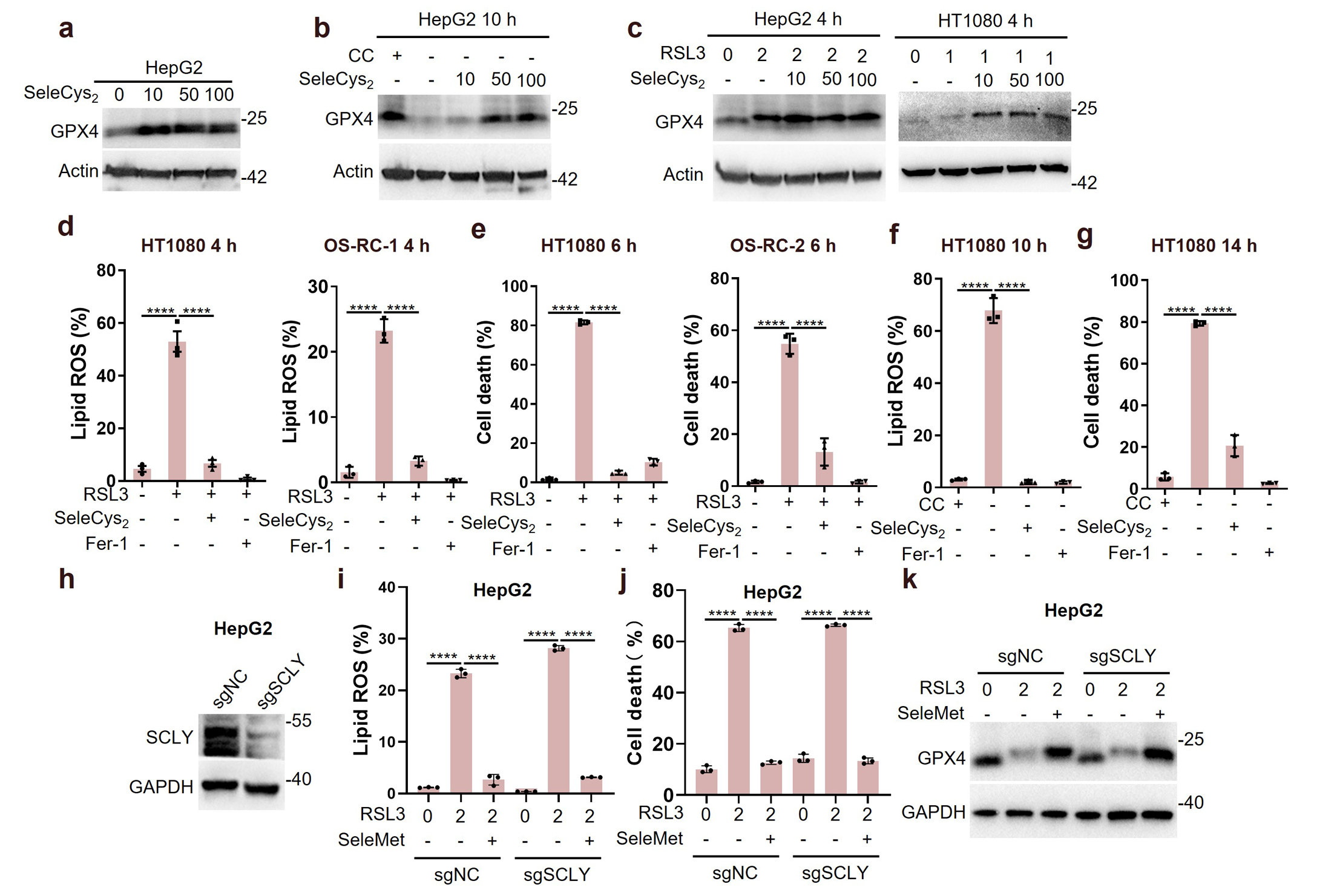

Supplement: Supplementary file 2 — Supplementary figure S1 [file 41419_2026_8466_MOESM2_ESM.tif]

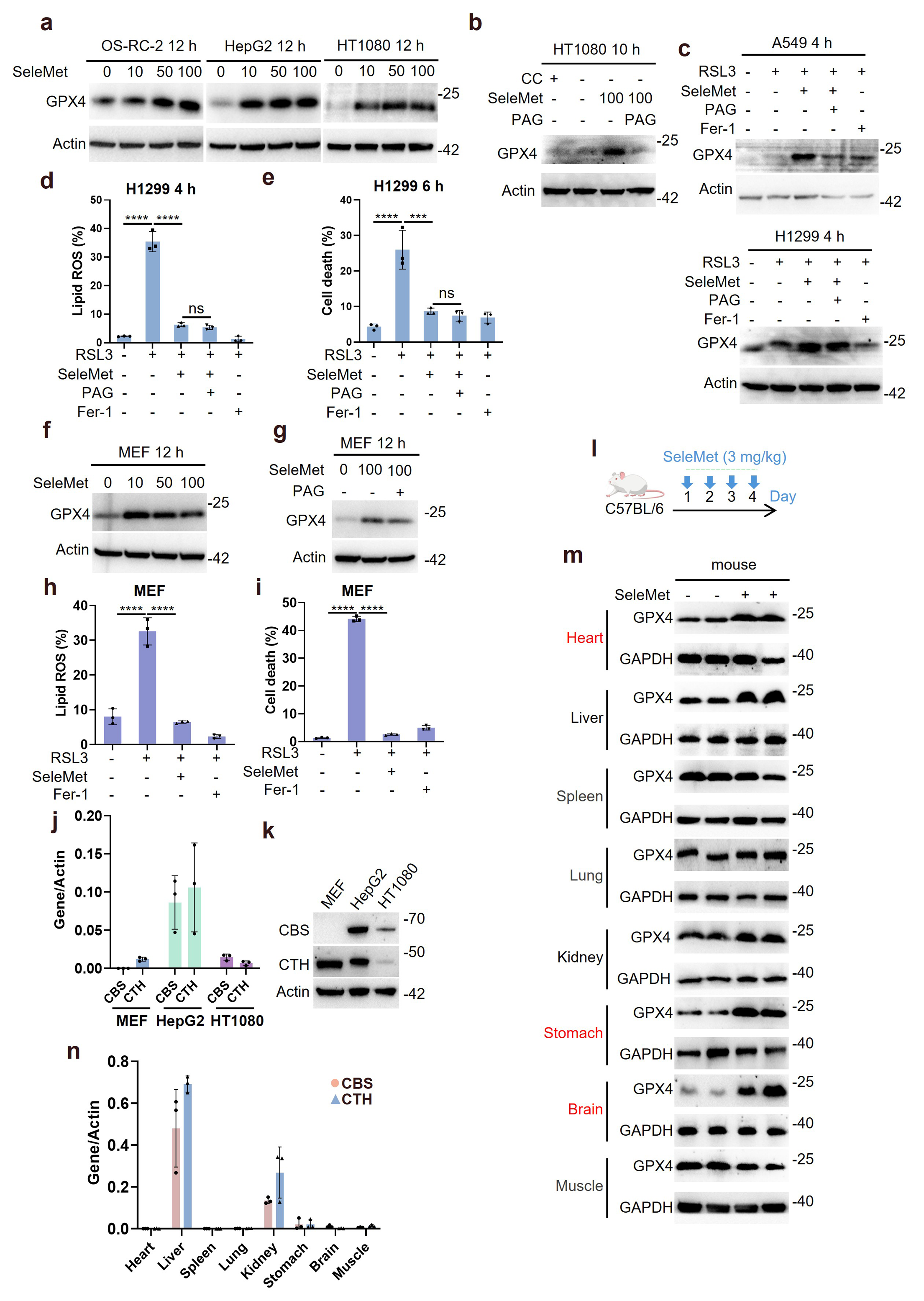

Supplement: Supplementary file 3 — Supplementary figure S2 [file 41419_2026_8466_MOESM3_ESM.tif]

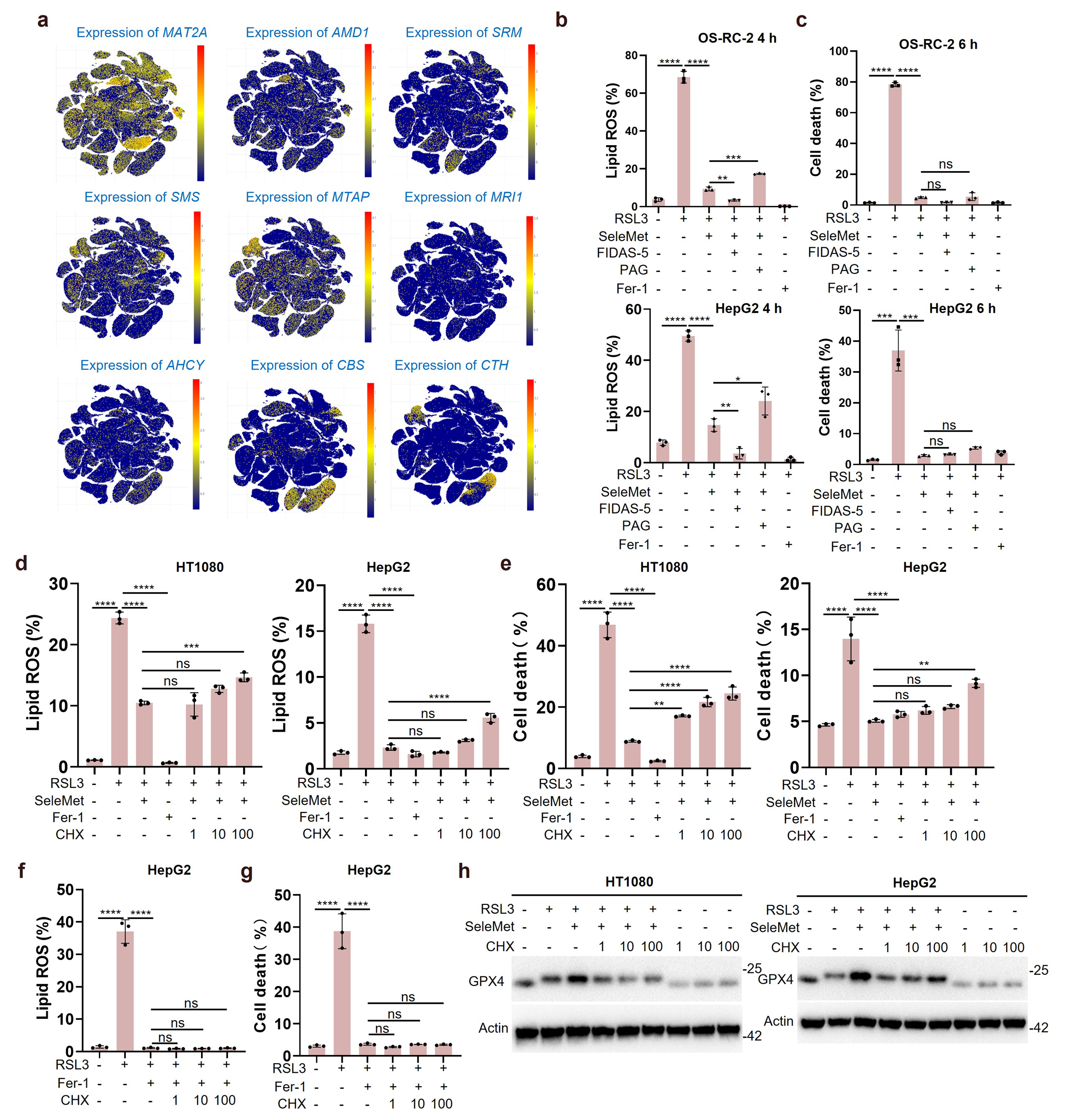

Supplement: Supplementary file 4 — Supplementary figure S3 [file 41419_2026_8466_MOESM4_ESM.tif]

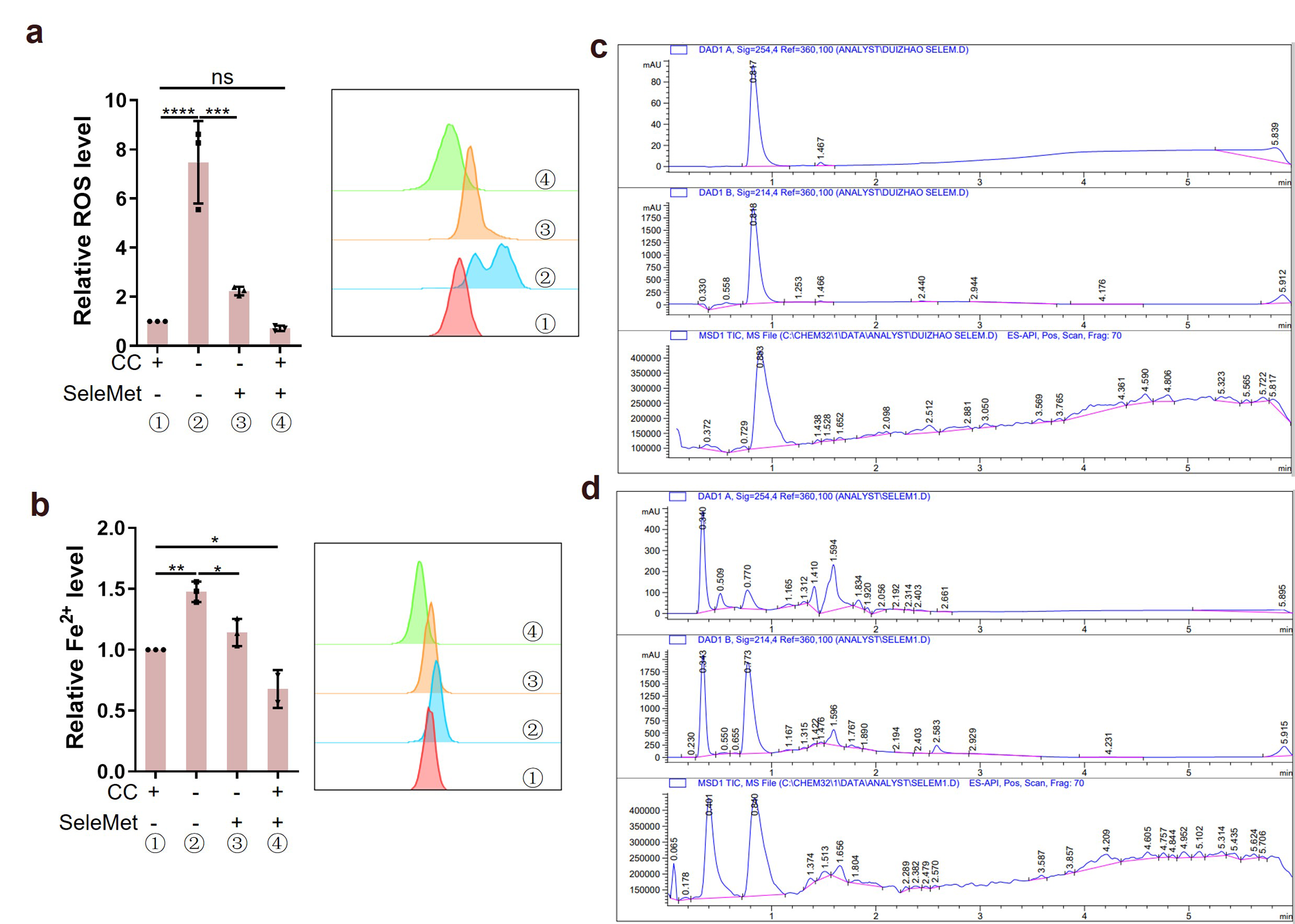

Supplement: Supplementary file 5 — Supplementary figure S4 [file 41419_2026_8466_MOESM5_ESM.tif]

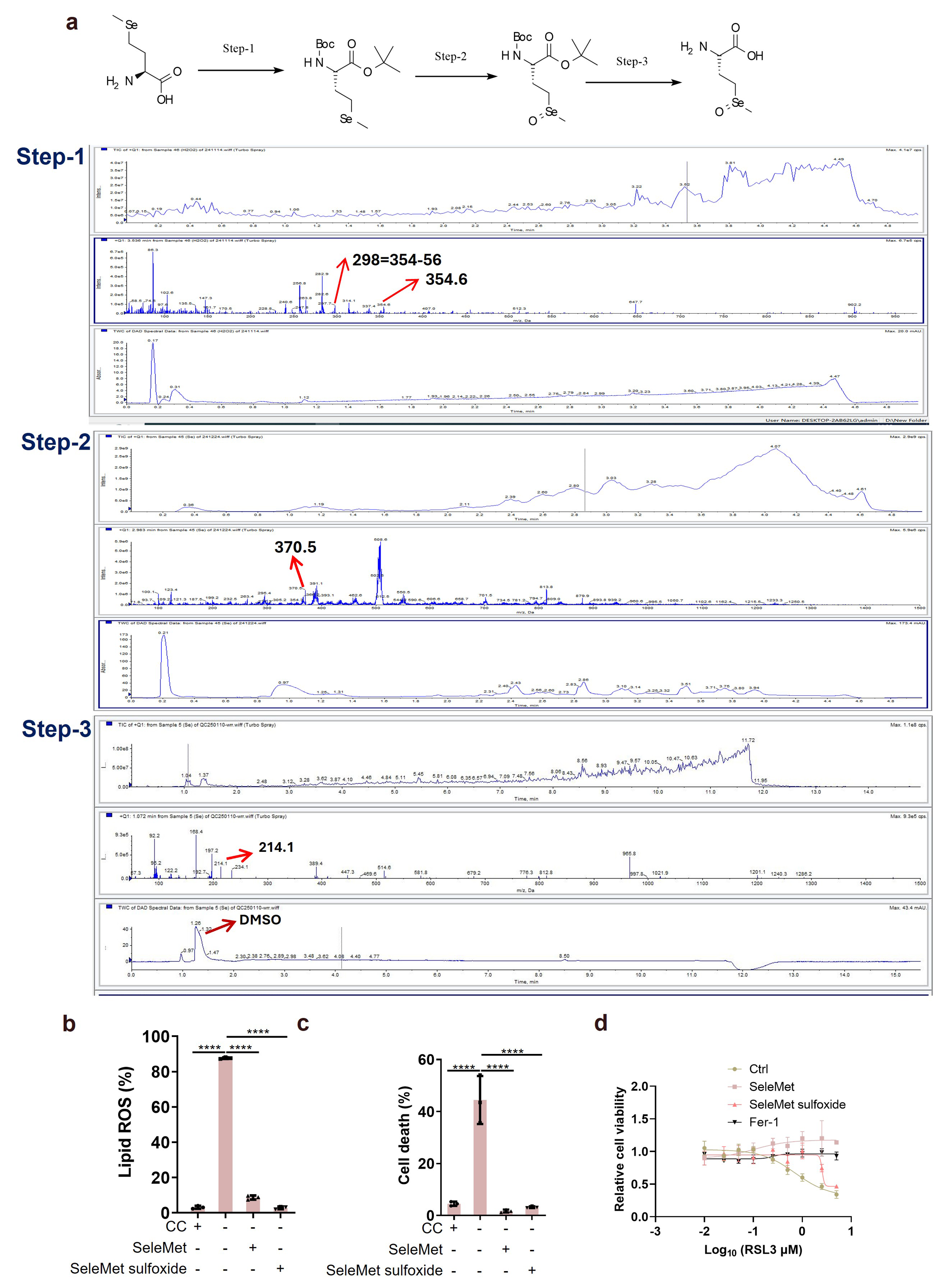

Supplement: Supplementary file 6 — Supplementary figure S5 [file 41419_2026_8466_MOESM6_ESM.tif]

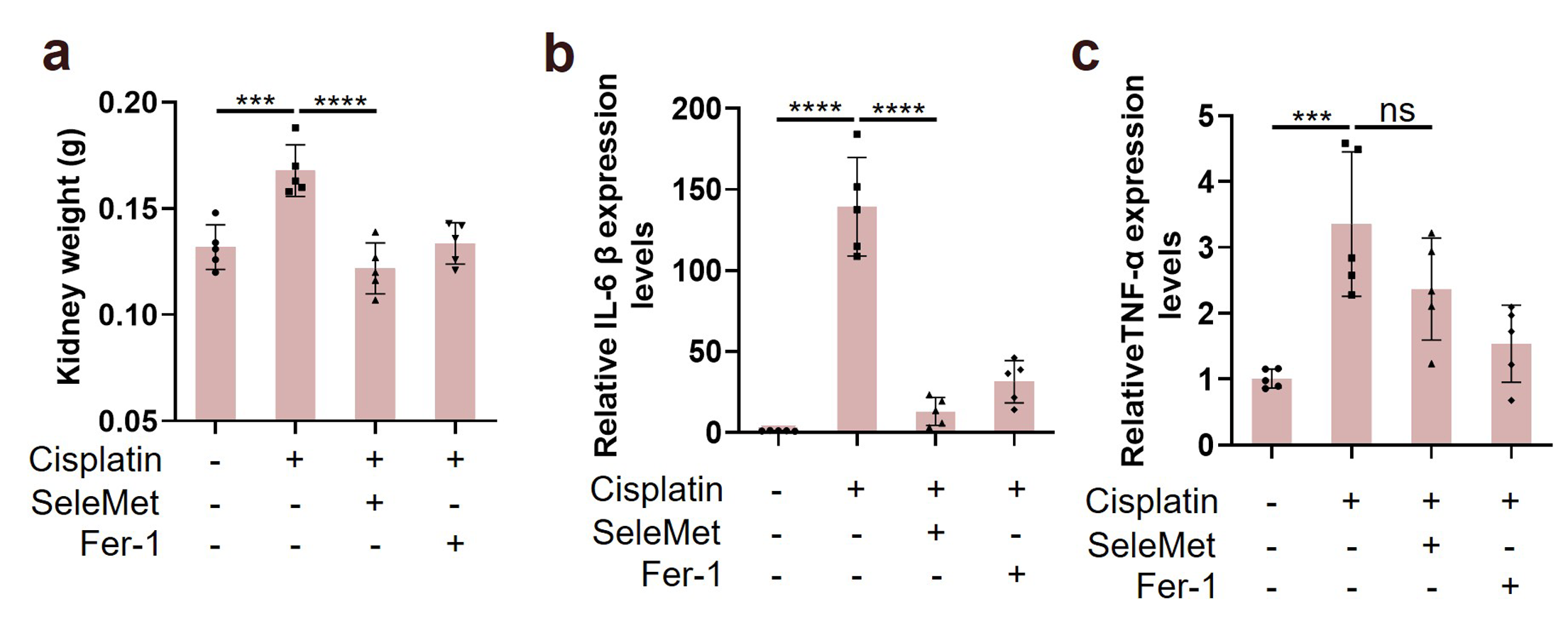

Supplement: Supplementary file 7 — Supplementary figure S6 [file 41419_2026_8466_MOESM7_ESM.tif]
